# Supplementary material for: Mice lacking DYRK2 exhibit congenital malformations with lung hypoplasia and altered Foxf1 expression gradient
Source: Commun Biol. 2021 Oct 20;4:1204. doi: 10.1038/s42003-021-02734-6 (PMC8528819; doi:10.1038/s42003-021-02734-6)
Supplement: Supplementary file 2 — Supplementary Information [file 42003_2021_2734_MOESM2_ESM.pdf]

Supplementary Information

Mice lacking DYRK2 exhibit congenital malformations with lung hypoplasia and altered Foxf1 expression gradient

by Yogosawa et al.

Supplementary Figure 1

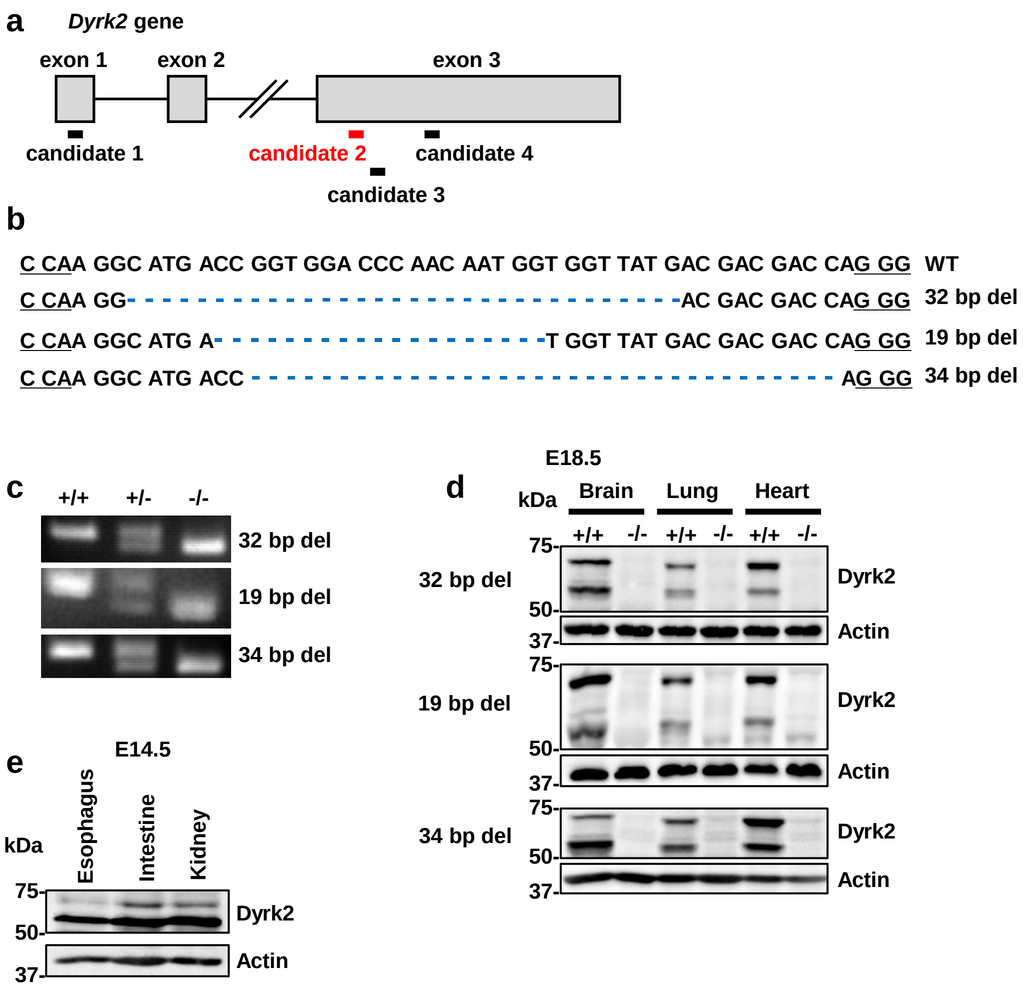

Supplementary Figure 1. Deletion of *Dyrk2* gene in C57BL/6J mice
a Schematic representation of the sgRNA used to target the mouse *Dyrk2* gene. **b** DNA sequencing analysis of the CRISPR target site in C57BL/6J WT and founder mice. Indels (deletions, dashed lines) were identified at the sgRNA-targeted loci. The PAM sequences are shown as underlined. **c** PCR-based genotyping of WT (+/+), *Dyrk2*$^{+/-}$ (+/-), and *Dyrk2*$^{-/-}$ (-/-) littermates. **d** Expression levels of Dyrk2 in the brain, lungs, and heart of the E18.5 WT and *Dyrk2*$^{-/-}$ mice. **e** Expression levels of Dyrk2 in the esophagus, intestine, and kidney of the E14.5 WT mice.

Supplementary Figure 2

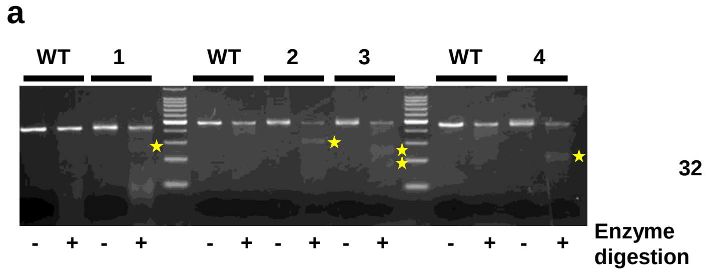

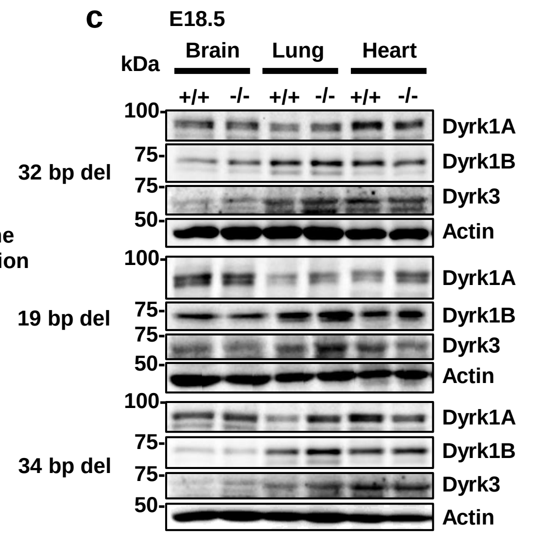

b

Sequence	Indel	Mouse
C CAA GGC ATG ACC GGT GGA CCC AAC AAT GGT GGT TAT GAC GAC GAC CAG GG	WT	
C CAA GG---------------------------T TAT GAC GAC GAC CAG GG	27 bp del	No.1
C CAA GG---------------------------T TAT GAC GAC GAC CAG GG	27 bp del	
C CAA GGC ATG ACC GGT GGA CCC AAC AAT GGT GGT TAT GAC GAC GAC ACGAC CAG GG	5 bp in	No.2
C CAA GGC ATG ---------------------------------GAC CAG GG	33 bp del	
C CAA GG --------------------------------AC GAC GAC CAG GG	32 bp del	No.3
C CAA GGC ------------------------------------GAC CAG GG	36 bp del	
C CAA GGC ------ GGT GGA CCC AAC AAT GGT GGT TAT GAC GAC GAC CAG GG	6 bp del	No.11
C CAA GGC ATG A -------------------T GGT TAT GAC GAC GAC CAG GG	19 bp del	
C CAA GG ------------------------T GGT TAT GAC GAC GAC CAG GG	24 bp in	No.12
C CAA GGC ATG ACC ----------------------------------AG GG	34 bp del	
C CAA GGC ATG ACC GG ---------------T GGT TAT GAC GAC GAC CAG GG	15 bp del	No.15
C CAA GGC ATG ACC GG ---------------T GGT TAT GAC GAC GAC CAG GG	15 bp del	
C CAA GG ------------------------T GGT TAT GAC GAC GAC CAG GG	24 bp in	No.16
C CAA GG --	45 bp del	
C CAA GGC ATG TG ACC GGT GGA CCC AAC AAT GGT GGT TAT GAC GAC GAC CAG GG	2 bp in	No.17
C CAA GGC ATG ACC GGT GGA CCC AAC AAT GG----- AT -- C G -- TAC CAG GG	9 bp del	
C CAA GGC A ---------------------------------AC GAC CAG GG	33 bp in	No.18
C CA ---G GG	45 bp del	

Supplementary Figure 2. Generation of *Dyrk2*$^{-/-}$ mice by CRISPR/Cas9 nickase system

a Gene editing efficiency of four candidate sgRNAs was determined using the GeneArt genomic cleavage detection assay in ES cell from C57BL/6J mice. Asterisks indicate cleavage fragment by enzyme digestion. **b** Indel mutations of founder mice generated by CRISPR/Cas9 nickase system. DNA sequencing analysis of the CRISPR target site in the C57BL/6J WT and founder mice. Indels (insertions, red characters; deletions, dashed lines) were identified at the sgRNA targeted loci. The PAM sequences are shown as underlined. Three mutated alleles (32, 19, and 34 bp del, blue characters) were observed in the founder mice. **c** Expression levels of Dyrk family (Dyrk1A, 1B, 3) in the brain, lung, and heart of the E18.5 WT and *Dyrk2*$^{-/-}$ mice.

Supplementary Figure 3

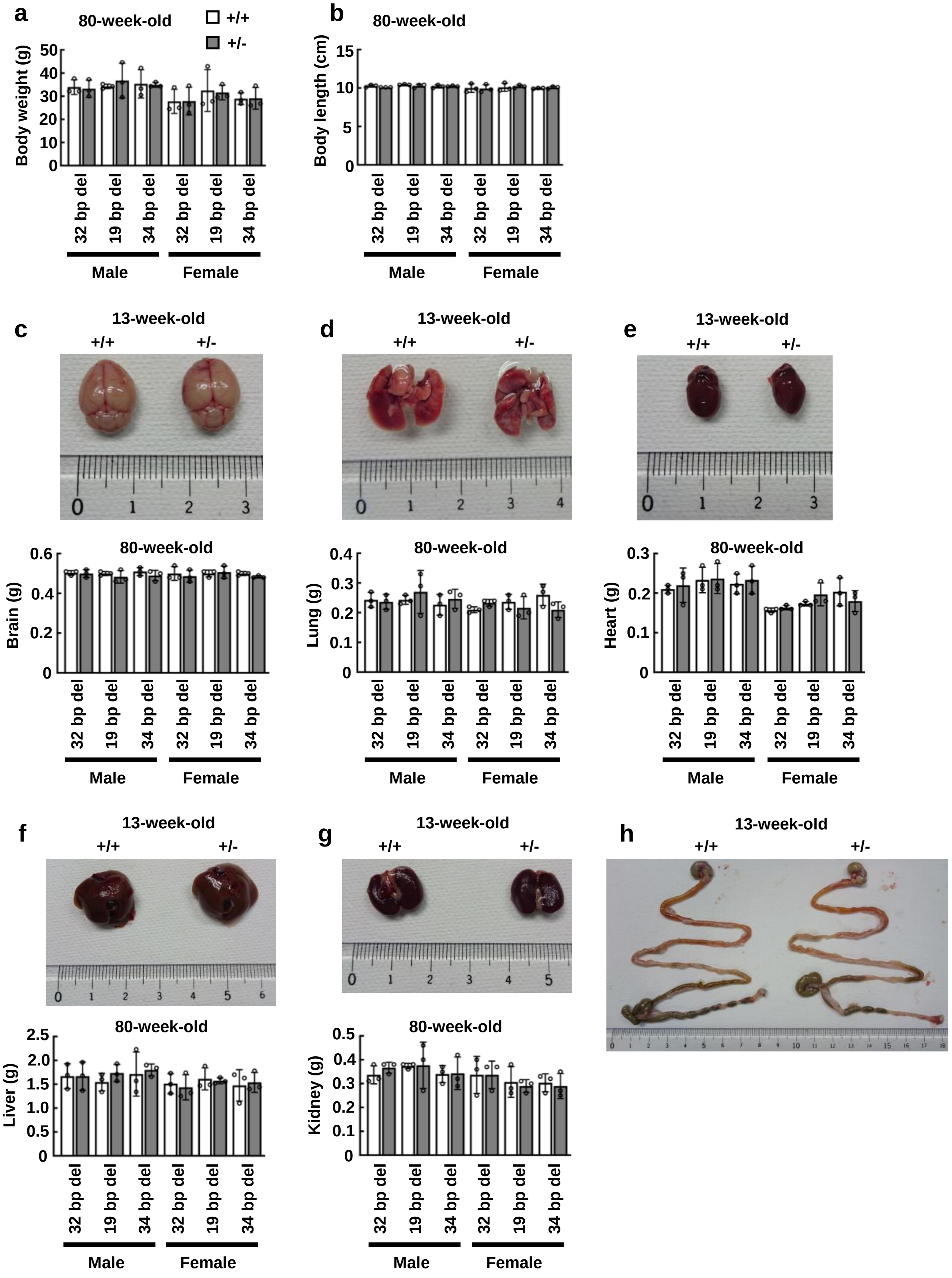

Supplementary Figure 3. ***Dyrk2*$^{+/-}$ mice show no significant difference**

a-b Body weight (**a**), body length (**b**) in 80-week-old WT and Dyrk2$^{+/-}$ mice. **c-g** Gross morphology (13-week-old) and weight (80-week-old) of brain (**c**), lung (**d**), heart (**e**), liver (**f**), and kidney (**g**) in WT (n = 3) and Dyrk2$^{+/-}$ mice (n = 3). **h** Gross morphology of intestine in 13-week-old WT and Dyrk2$^{+/-}$ mice.

Supplementary Figure 4

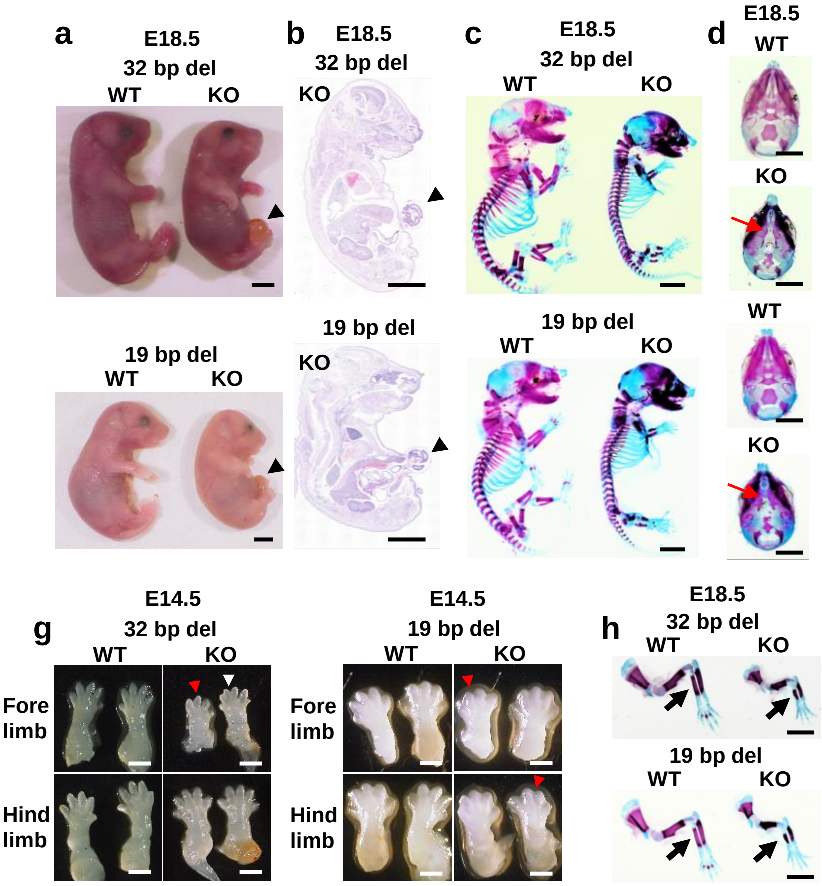

Supplementary Figure 4. Loss of Dyrk2 causes multiple developmental abnormalities
a Lateral views of E18.5 embryos. Arrowheads; omphalocele. **b** Lateral views of H&E sections in E18.5 embryos. Arrowheads; omphalocele. **c** Lateral views of skeleton preps in E18.5 embryos. **d** Palatal shelves in E18.5 embryos. Red arrows; cleft palates. **e** H&E staining of palate in E18.5 embryos. Red arrowheads; cleft palates. T, tongue. **f** H&E staining of skin in E18.5 embryos. Numbers denote the stages of hair follicle morphogenesis. **g** Limb dysmorphology of E14.5 embryos. The red arrowheads; ectrodactyly. The white arrowhead; syndactyly and polydactyly. **h** Radial anomalies of E18.5 embryos. Black arrows; radial bones. Scale bar: 3 mm in (**a, b, c, d, h**), 400 μm in (**e**), 200 μm in (**f**), 1 mm in (**g**).

Supplementary Figure 5

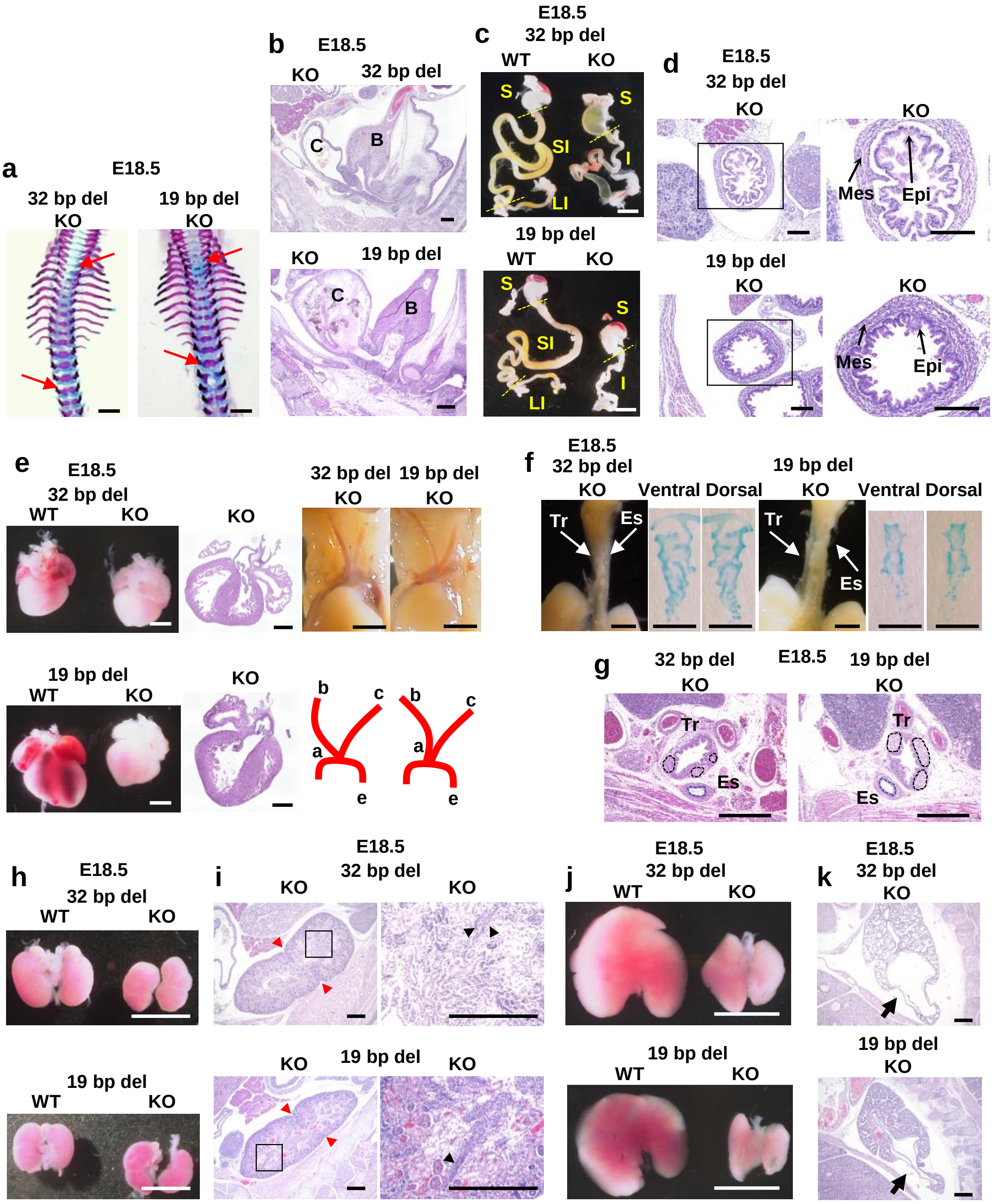

Supplementary Figure 5. Loss of Dyrk2 causes multiple developmental abnormalities
a Spine defects of E18.5 embryos. Red arrows; the lack of vertebral bodies and butterfly vertebrae. **b** H&E staining of the anus and cloaca in E18.5 embryos. B, bladder; C, cloaca. **c** Gross morphology of intestine in E18.5 embryos. S, stomach; SI, small intestine; LI, large intestine; I, intestine. **d** H&E staining of intestine in E18.5 embryos. Epi, epithelium; Mes, mesenchyme. **e** Gross morphology and H&E staining of the heart and cardiac outflow tract in E18.5 embryos. Insets in each frame is a schematic of the aortic arch with the aorta and tributaries. a, ascending aorta; b, brachiocephalic artery; c, left common carotid artery; e, descending aorta. **f** Gross morphology of trachea (Tr) and esophagus (Es), and alcian blue staining of cartilaginous rings in E18.5 embryos. **g** H&E staining of trachea (Tr) and esophagus (Es) in E18.5 embryos. Dashed black lines; cartilaginous rings. Dashed blue lines; esophagus. **h** Gross morphology of kidneys in E18.5 embryos. **i** H&E staining of kidneys in E18.5 embryos. Red arrowheads; the lobe folds. Black arrowheads; medullary collecting ducts. **j** Gross morphology of lungs in E18.5 embryos. **k** H&E staining of lungs in E18.5 embryos. Black arrows; lung cysts. Scale bar: 1.5 mm in (**a**), 3 mm in (**c, h, j**), 400 μm in (**b, g, i, k**), 200 μm in (**d**), 1 mm in (**e, f**).

Supplementary Figure 6

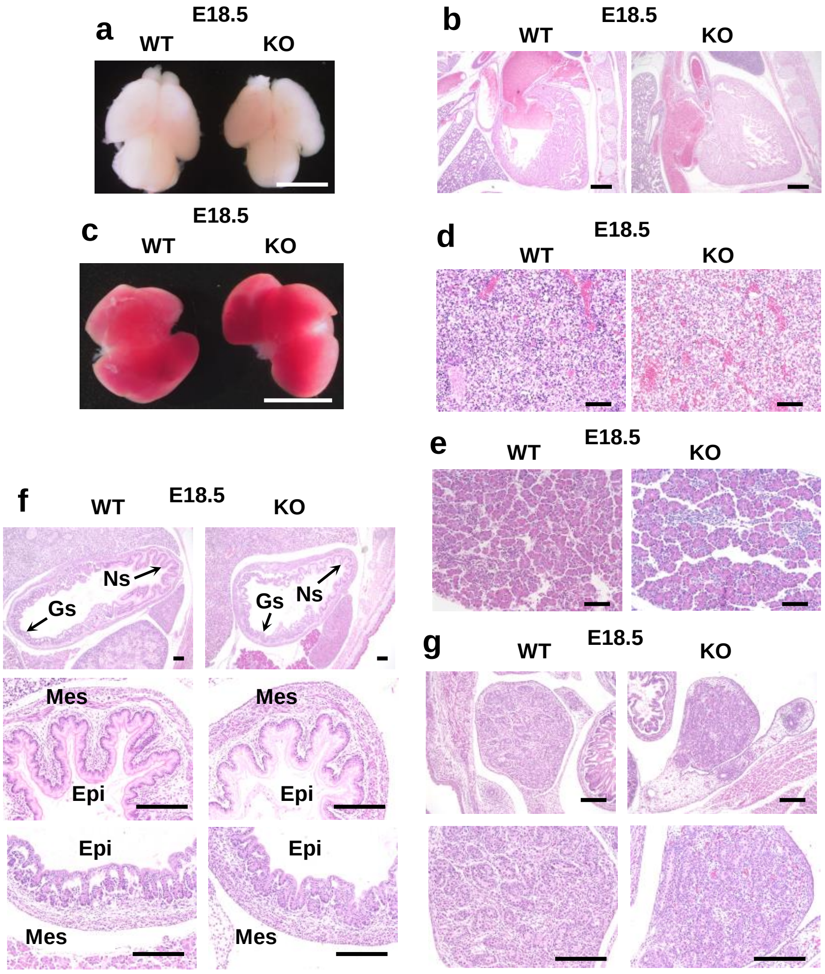

Supplementary Figure 6. ***Dyrk2****$^{-/-}$* **embryos showed no morphological abnormalities**
a Gross morphology of brain in E18.5 embryos. **b** H&E staining of heart in E18.5 embryos. **c** Gross morphology of liver in E18.5 embryos. **d** H&E staining of liver in E18.5 embryos. **e-g** H&E staining of pancreas (**e**), stomach (**f**), testis (**g**) in E18.5 embryos. Epi, epithelium; Mes, mesenchyme; Gs, glandular stomach; Ns, non-glandular stomach. Scale bar: 3 mm in (**a**, **c**), 400 μm in (**b**), 100 μm in (**d, e**), and 200 μm in (**f, g**).

Supplementary Figure 7

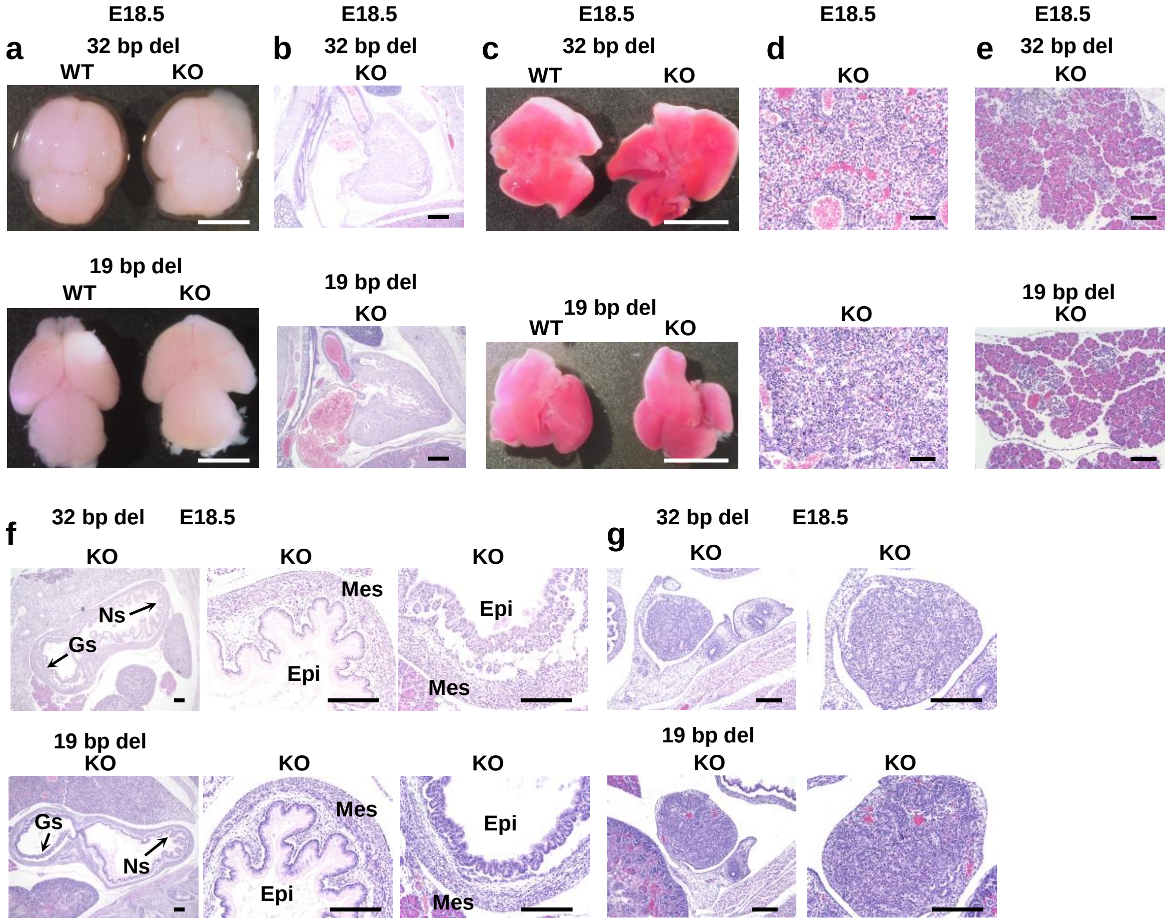

Supplementary Figure 7. ***Dyrk2*$^{-/-}$ embryos showed no morphological abnormalities**
a Gross morphology of brain in E18.5 embryos. **b** H&E staining of heart in E18.5 embryos. **c** Gross morphology of liver in E18.5 embryos. **d** H&E staining of liver in E18.5 embryos. **e-g** H&E staining of pancreas (**e**), stomach (**f**), testis (**g**) in E18.5 embryos. Epi, epithelium; Mes, mesenchyme; Gs, glandular stomach; Ns, non-glandular stomach. Scale bar: 3 mm in (**a**, **c**), 400 μm in (**b**), 100 μm in (**d, e**), and 200 μm in (**f, g**).

Supplementary Figure 8

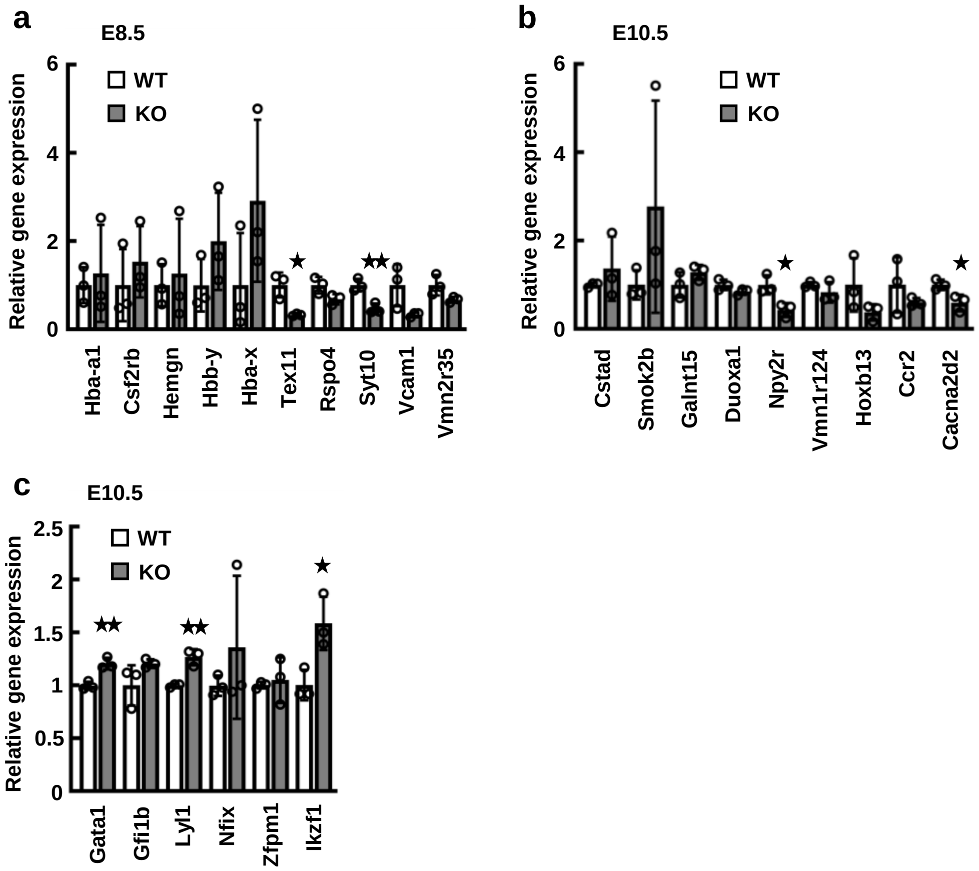

Supplementary Figure 8. The expression levels of genes from the microarray data

a, b Relative expression of top differentially expressed genes from the microarray data in E8.5 (**a**) and 10.5 (**b**) (n = 3). **c** Relative expression of genes related to lymphocyte and erythrocyte development in E10.5 (n = 3). Data are presented as the mean ± SD. *p < 0.05; **p < 0.01.

Supplementary Figure 9

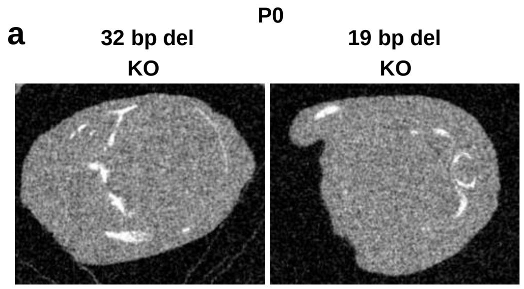

Supplementary Figure 9. *Dyrk2$^{-/-}$* mice show respiratory failure
a Micro-CT analysis of the lung from P0 *Dyrk2$^{-/-}$* pups.

Supplementary Figure 10

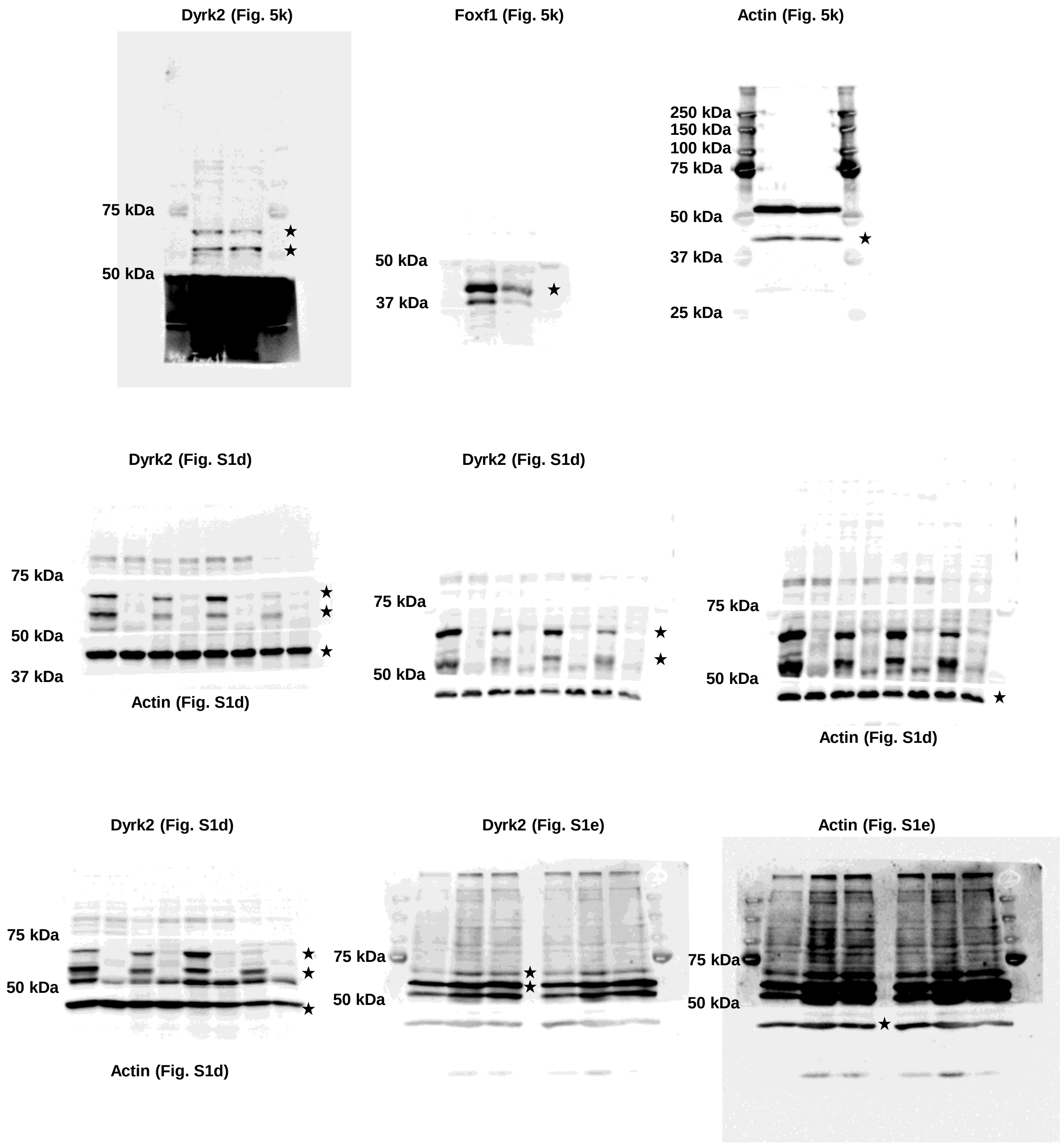

Supplementary Figure 10 continued

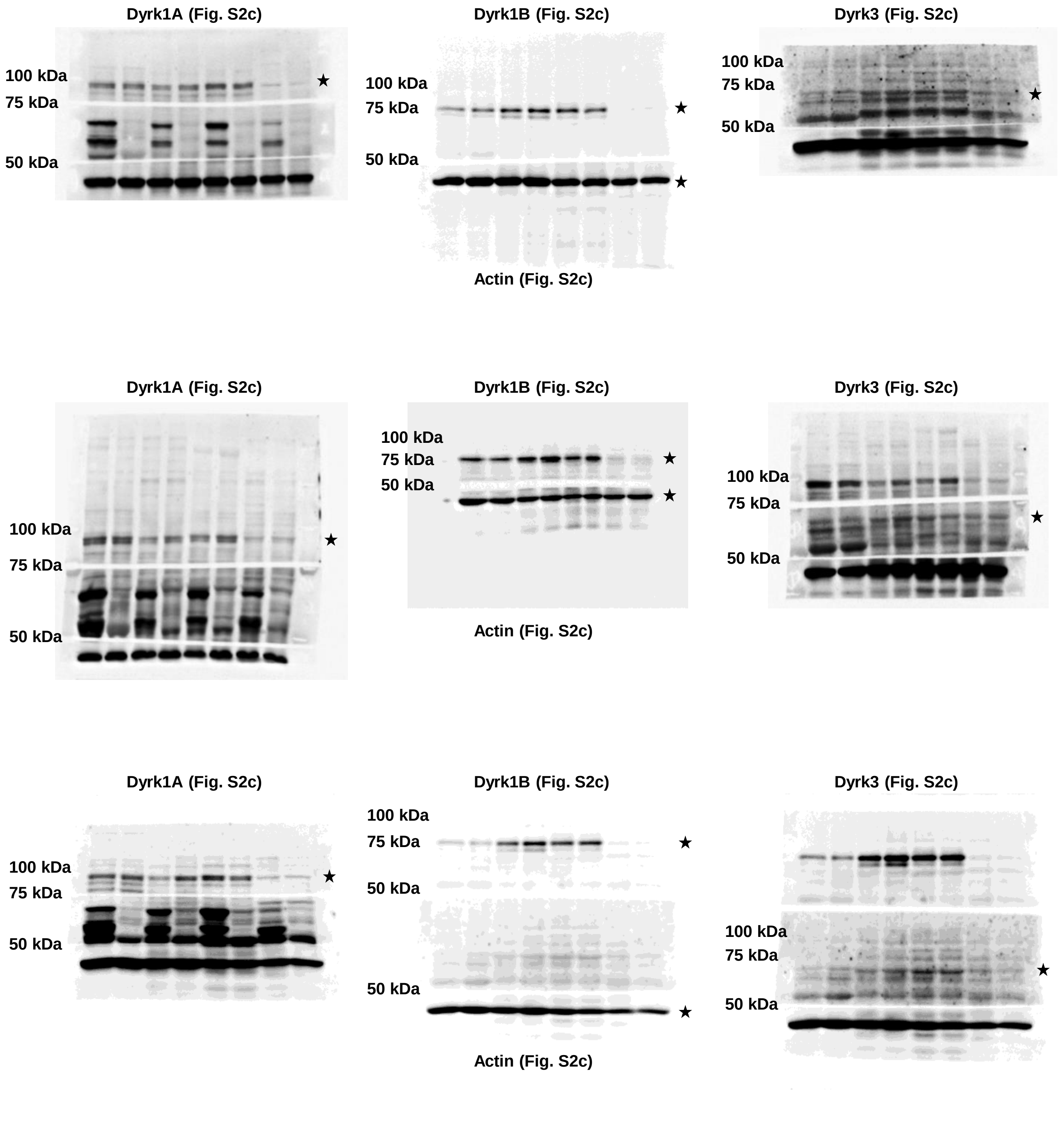

Supplementary Figure 10. Uncropped images of the immunoblots from Fig. 5k and Supplementary Fig. 1d, e, 2c. The asterisks highlight the bands shown in Fig. 5k and Supplementary Fig. 1d, e, 2c.

Supplementary Figure 11

32 bp del (Fig. S1c)

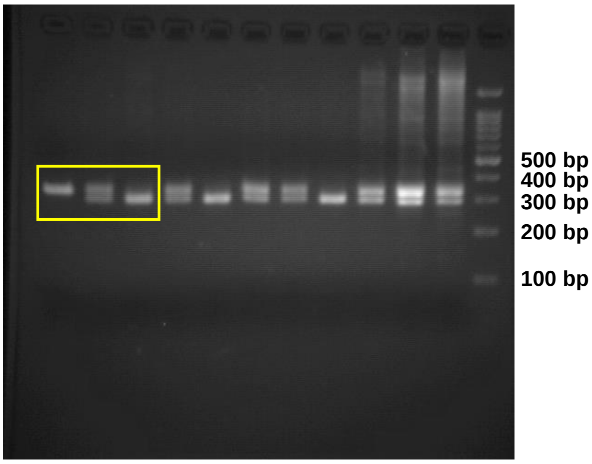

19 bp del (Fig. S1c)

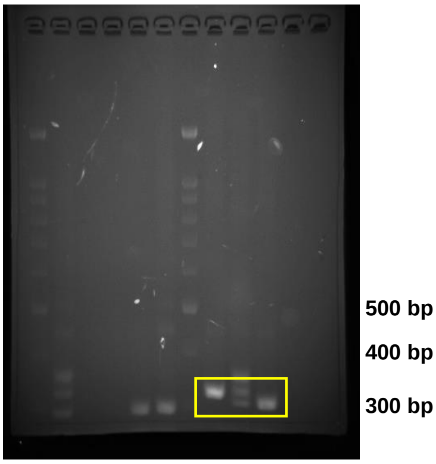

34 bp del (Fig. S1c)

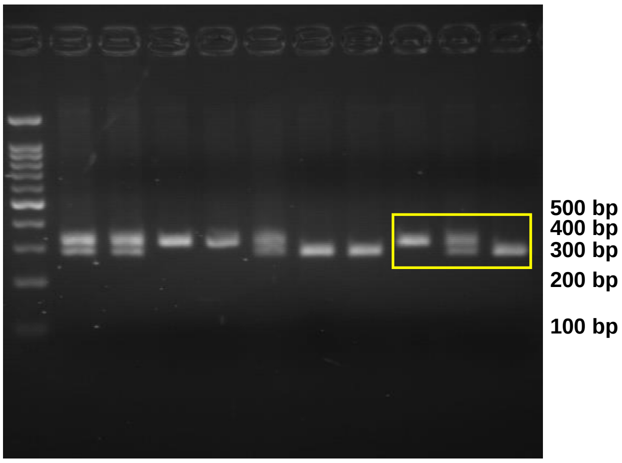

Fig. S2a

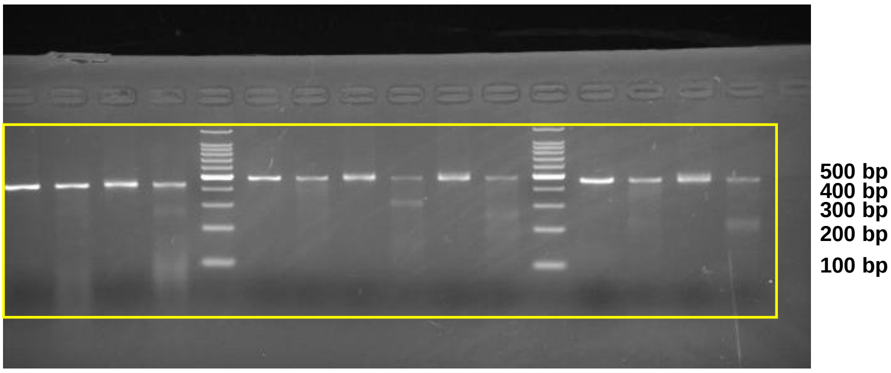

Supplementary Figure 11. Uncropped images of the gels from Supplementary Fig. 1c, 2a.

Supplementary Table 1. Sequence for paired single guide RNAs (sgRNAs) and genotyping primer

Candidate	Target	Sequence
Candidate 1	Forward<br>Reverse	CCGCGGGAGAGGAACGTGCATGG<br>CATGTTAACCAGGAAACCTTCGG
Candidate 2	Forward<br>Reverse	TGGGTCCACCGGTCATGCCTTGG<br>GGTGGTTATGACGACGACCAGGG
Candidate 3	Forward<br>Reverse	GCACCTGCACGTACGATCCCTGG<br>TTACAGGTACGAGGTCCTCAAGG
Candidate 4	Forward<br>Reverse	CGAGGATCACTTCCGGAGCCCGG<br>ATGGCATGCCCATAGACATGTGG
Primer for genotyping		
Gene	Primer	Sequence
Dyrk2	Forward<br>Reverse	CTCTCTGAAGTCCATGGAAGG<br>GTGCTGGTGGACTTTGTGGT

Supplementary Table 2. Indel mutation of F0 pups

No. of pups	Indel mutation
No.1	27 bp and 27 bp deletion
No.2	5 bp insertion and 33 bp deletion
No.3	32 bp and 36 bp deletion
No.4	WT
No.11	6 bp and 19 bp deletion
No.12	24 bp and 34 bp deletion
No.13	WT
No.14	WT
No.15	15 bp and 15 bp deletion
No.16	24 bp and 45 bp deletion
No.17	2 bp insertion and 9 bp deletion and a mutation
No.18	33 bp and 45 bp deletion
No.19	WT

Supplementary Table 3. Survival of *Dyrk2*$^{-/-}$ mice

**32 bp del**	+/+	+/-	-/-			
E18.5	20	44	23			
P0	6	12	0 (7)			
4W	27	84	0			
**19 bp del**	+/+	+/-	-/-			
E18.5	15	38	13			
P0	5	16	0 (7)			
4W	35	56	0			
**34 bp del**	+/+	+/-	-/-			
E18.5	44	71	34			
P0	7	6	0 (1)			
4W	39	104	0			
**Total (all deletion patterns)**	+/+	+/-	-/-	**Total**	**χ^2 2df**	**P**
E18.5	79 (75.5)	153 (151)	70 (75.5)	302	0.589	0.74
P0	18 (16.75)	34 (33.5)	0 (16.75)	67	16.851	0.0002
4W	101 (86.25)	244 (172.5)	0 (86.25)	345	118.409	0

Actual and expected numbers and χ^2 analysis of progeny from intercrosses of *Dyrk2*$^{+/-}$ mice with 2 degrees of freedom. The number of dead neonates in each deletion pattern is shown in parentheses. In total column, expected numbers for each genotype based on Mendelian ratios of 1:2:1 are indicated in parentheses. P is the statistical significance value.

Supplementary Table 4. Primers for quantitative PCR

Gene	Primer	Sequence
Zic3	Forward	cctgcgcaaacacatgaa
	Reverse	ctatagcgggtggagtggaa
Foxa2	Forward	aagtagccaccacacttcagg
	Reverse	tgtggcccatctatttaggg
Sox9	Forward	gtacccgcatctgcacaac
	Reverse	ctcctccacgaagggtctct
Notch1	Forward	ggatgctgactgcatggat
	Reverse	aatcatgaggggtgtgaagc
Pax1	Forward	cggacgtttatggagcaaac
	Reverse	tccatcttgggggagtagg
Scx	Forward	gagaacacccagcccaaac
	Reverse	ttctgtcacggtctttgctc
Hoxd12	Forward	tcttgcctgcgatcttcactg
	Reverse	accaggaattcgttctccagct
Hoxd13	Forward	ggaacagccaggtgtactgtg
	Reverse	ggctggtttaaagccacatc
Foxf2	Forward	gcagagctacttgcaccaga
	Reverse	ttgaggacgaaatctttcctgt
Foxl1	Forward	ccatgaagaagggacaaagc
	Reverse	ccaccggggagtcctaag
Cdx2	Forward	caccatcaggaggaaaagtga
	Reverse	ctgcggttctgaaaccaaat
Nkx2.1	Forward	aaaactgcggggatctgag
	Reverse	tgctttggactcatcgacat
Foxp2	Forward	gacccctggagaggactaaag
	Reverse	cttggagtggagtcctaagttca
Brachyury	Forward	cagcccacctactggctcta
	Reverse	gagcctggggtgatggta
Tbx4	Forward	ctgcatgagaaggagctgtg
	Reverse	ttgtagctggggaacatcct

Tbx5	Forward<br>Reverse	ggatgtctcggatgcaaagt<br>ggttggaggtgactttgtgc
Fgf10	Forward<br>Reverse	cgggaccaagaatgaagact<br>gcaacaactccgatttccac
Fgfr2b	Forward<br>Reverse	gggataaatagctccaatgc<br>tcacaggcgcttgctgtttg
Shh	Forward<br>Reverse	ccaattacaaccccgacatc<br>gcatttaacttgtctttgcacct
Foxf1	Forward<br>Reverse	agcatctccacgcactcc<br>tgtgagtgataccgagggatg
Podoplanin	Forward<br>Reverse	gccagtgttgttctgggttt<br>tctcctgtacctggggtcac
SP-C	Forward<br>Reverse	ggtcctgatggagagtccac<br>gatgagaaggcgtttgaggt
Vegfr2	Forward<br>Reverse	ccccaaattccattatgacaa<br>cggctctttcgcttactgtt
αSMA	Forward<br>Reverse	taacccttcagcgttcagc<br>acatagctggagcagcgtct
Myocd	Forward<br>Reverse	gcaagggcagaaacaggtc<br>atctgagcagttggaatggac
Hoxb7	Forward<br>Reverse	ctggatgcgaagctcagg<br>ccgagtcaggtagcgattgta
Wif1	Forward<br>Reverse	ccaaggagacctgtgctctaa<br>tctcgacactggcacttgtt
Hba-a1	Forward<br>Reverse	tgacagactcaggaagaaacca<br>gggaagctagcaaacatcctt
Csf2rb	Forward<br>Reverse	atctgggcatccagctcat<br>agctgatgctgacgttcttg
Hemgn	Forward<br>Reverse	cctgaagctcacccacaga<br>tctcagactccaagatccaatg
Hbb-y	Forward<br>Reverse	tggtgaagccttgggaag<br>ctttgacccttgggttgc
Hba-x	Forward<br>Reverse	catcatgtccatgtgggaga<br>ggggtagctgcagaagagc

Tex11	Forward<br>Reverse	tgcacataaggccatgaaaa<br>ttgtgcatgcatacgctgt
Rspo4	Forward<br>Reverse	tctgagtccaggaagtgtcctat<br>tgttcttctgcctgggattt
Syt10	Forward<br>Reverse	catctttccagccgatagga<br>cgctaacaggctgactgaaa
Vcam1	Forward<br>Reverse	tcttacctgtgcgctgtgac<br>actggatcttcagggaatgagt
Vmn2r35	Forward<br>Reverse	gaggtggccacaggaagtaaaa<br>caggggctgcaaacagaaca
Cstad	Forward<br>Reverse	tcccagctactcaggcaatc<br>caggtcacccaagagatgct
Smok2b	Forward<br>Reverse	acgtggggcacagtagagtc<br>ggcatcctctggtgatcttc
Galnt15	Forward<br>Reverse	gcccctcctcagcagaat<br>cacgtctatgactggagacacc
Duoxa1	Forward<br>Reverse	gaagcagaattcaagctctgg<br>aagggcagtgtgtgtccaa
Npy2r	Forward<br>Reverse	acgcgcaagagtcaatacag<br>ccatagggctccactttcac
Vmn1r124	Forward<br>Reverse	ttctcttctcttcatgaggcttg<br>aaagtgcagatactggggaaga
Hoxb13	Forward<br>Reverse	ggaaagcagcgtttgcag<br>ttgctatagggaatgcgttttt
Ccr2	Forward<br>Reverse	acctgtaaatgccatgcaagt<br>tgtcttccatttcctttgatttg
Cacna2d2	Forward<br>Reverse	cagcaaactgaaggattttgag<br>ttcaagtccttgcaatactctctc
Gata1	Forward<br>Reverse	ccctgaactcgtcataccact<br>gaacactggggttgaacctg
Gfi1b	Forward<br>Reverse	gttgctgaaccagagccttc<br>ttggggtgtcacgagagg
Lyl1	Forward<br>Reverse	gcaggacccttcagcatct<br>ccaccttctggggttggt

Nfix	Forward<br>Reverse	tgtttgggtctttcgctgtt<br>cgaaattgttcccaagttgc
Zfpm1	Forward<br>Reverse	gcccctgcagagttctttt<br>aaggtttcactctctctagtagtctgc
Ikzf1	Forward<br>Reverse	tgccacaactacttggaaagc<br>ctctctgctcctatcttgcaca
36B4	Forward<br>Reverse	ggccctgcactctcgctttc<br>tgccaggacgcgcttgt

Supplementary Table 5. Antibody list

Antibody	Dilution	Company,<br>Catalog code
DYRK2	1:1000 (WB)<br>1:3000 or 1:1000 (IHC)	SIGMA<br>HPA027230
DYRK1A	1:1000 (WB)	Cell Signaling Technology<br>#2771
DYRK1B	1:1000 (WB)	Cell Signaling Technology<br>#5672
DYRK3	1:1000 (WB)	SIGMA<br>SAB2105219
Actin	1:2000 (WB)	Lab vision<br>MS-1295-P0
FoxF1	1:1000 (WB)<br>1:200 (IHC)	R&D systems<br>AF4798
E-Cadherin	1:400 (IHC)	Cell Signaling Technology<br>#3195
Podoplanin	1:500 (IHC)	abcam<br>ab11936
Prosurfactant Protein C	1:1000 (IHC)	abcam<br>ab90716
VEGFR2	1:50 (IHC)	Cell Signaling Technology<br>#2479
CC10	1:400 (IHC)	Santa Cruz<br>sc-390313
FoxJ1	1:50 (IHC)	Thermo Fisher Scientific<br>14-9965-82
Acetylated Tubulin	1:3000 or 1:5000 (IHC)	SIGMA<br>T6793
Ki67	1:500 (IHC)	BD Biosciences<br>550609
Cleaved Caspase-3	1:1000 (IHC)	Cell Signaling Technology<br>#9664

Supplementary Table 6. GO analysis

**GO analysis E8.5**		
GO term	Count	P_value
Homeobox	28	2.40E-06
Oxygen transport	8	5.64E-07
EGF-like domain	24	4.64E-06
Transcription regulation	96	4.25E-05
Transcription	97	9.24E-05
Blood coagulation	6	0.0186205
Hemostasis	6	0.0186205
Collagen	8	0.0301141
Heme	14	0.0076063
Iron	17	0.2513488
**GO analysis E10.5**		
GO term	Count	P_value
Homeobox	27	2.09E-08
Oxygen transport	6	3.90E-05
Heme biosynthesis	4	0.00505
Porphyrin biosynthesis	3	0.0168293
Actin capping	3	0.0701867
Transcription regulation	65	0.0056317
Transcription	65	0.0117723
Voltage-gated channel	10	0.0089154
Sodium transport	8	0.0274232
Sodium	8	0.0363235
