# Supplementary material for: Mice lacking DYRK2 exhibit congenital malformations with lung hypoplasia and altered Foxf1 expression gradient
Source: Commun Biol. 2021 Oct 20;4:1204. doi: 10.1038/s42003-021-02734-6 (PMC8528819; doi:10.1038/s42003-021-02734-6)
Supplement: Supplementary file 3 — Description of Additional Supplementary Files [file 42003_2021_2734_MOESM3_ESM.pdf]

Description of Additional Supplementary Files

File name: Supplemental Movie 1

Description: *Respiratory movements involving the whole-body muscles immediately after birth in WT mice.* Normal respiratory movement was observed in E18.5 WT mice.

File name: Supplemental Movie 2

Description: *Respiratory movements involving the whole-body muscles immediately after birth in Dyrk2$^{-/-}$ mice.* The E18.5 *Dyrk2*$^{-/-}$ mice were shown in deep respiratory movements involving the whole-body muscles but failed to initiate normal breathing.

File name: Supplementary Data 1

Description: *Summary of the abnormal phenotypes in Dyrk2$^{-/-}$ mice.* The summary of abnormal phenotypes in 3 different *Dyrk2*$^{-/-}$ mice lines. Detailed abnormal phenotypes of each organ are indicated in parentheses.

File name: Supplementary Data 2

Description: *Source data underlying plots shown in figures.* Each sheet shows the raw data for each individual figure. The sheets are named according to the figure number.
