# Supplementary material for: Mice lacking DYRK2 exhibit congenital malformations with lung hypoplasia and altered Foxf1 expression gradient
Source: Commun Biol. 2021 Oct 20;4:1204. doi: 10.1038/s42003-021-02734-6 (PMC8528819; doi:10.1038/s42003-021-02734-6)
Supplement: Supplementary file 8 — Reporting Summary [file 42003_2021_2734_MOESM8_ESM.pdf]

nature research

Corresponding author(s): Kiyotsugu Yoshida

Last updated by author(s): Sep 3, 2021

Reporting Summary

Nature Research wishes to improve the reproducibility of the work that we publish. This form provides structure for consistency and transparency in reporting. For further information on Nature Research policies, see our Editorial Policies and the Editorial Policy Checklist.

Statistics

For all statistical analyses, confirm that the following items are present in the figure legend, table legend, main text, or Methods section.

n/a	Confirmed	
☐	☒	The exact sample size (*n*) for each experimental group/condition, given as a discrete number and unit of measurement
☐	☒	A statement on whether measurements were taken from distinct samples or whether the same sample was measured repeatedly
☐	☒	The statistical test(s) used AND whether they are one- or two-sided<br>*Only common tests should be described solely by name; describe more complex techniques in the Methods section.*
☒	☐	A description of all covariates tested
☒	☐	A description of any assumptions or corrections, such as tests of normality and adjustment for multiple comparisons
☐	☒	A full description of the statistical parameters including central tendency (e.g. means) or other basic estimates (e.g. regression coefficient) AND variation (e.g. standard deviation) or associated estimates of uncertainty (e.g. confidence intervals)
☐	☒	For null hypothesis testing, the test statistic (e.g. *F*, *t*, *r*) with confidence intervals, effect sizes, degrees of freedom and *P* value noted<br>*Give P values as exact values whenever suitable.*
☒	☐	For Bayesian analysis, information on the choice of priors and Markov chain Monte Carlo settings
☒	☐	For hierarchical and complex designs, identification of the appropriate level for tests and full reporting of outcomes
☒	☐	Estimates of effect sizes (e.g. Cohen's *d*, Pearson's *r*), indicating how they were calculated

Our web collection on statistics for biologists contains articles on many of the points above.

Software and code

Policy information about availability of computer code

Data collection	PicoReal96, Chemiluminescence imaging system, Fluorescence microscope, Latheta LCT-200, Digital camera (Nikon, D5500)
Data analysis	Image J, DAVID Bioinformatics Resources 6.8, The Comprehensive R Archive Network with R (version 3.6.1), online calculation chi-square tool (http://www.quantpsy.org), GraphPad Prism 9.0.0

For manuscripts utilizing custom algorithms or software that are central to the research but not yet described in published literature, software must be made available to editors and reviewers. We strongly encourage code deposition in a community repository (e.g. GitHub). See the Nature Research guidelines for submitting code & software for further information.

Data

Policy information about availability of data

All manuscripts must include a data availability statement. This statement should provide the following information, where applicable:

- Accession codes, unique identifiers, or web links for publicly available datasets
- A list of figures that have associated raw data
- A description of any restrictions on data availability

Microarray data can be accessed through the Gene Expression Omnibus (GEO) under the NCBI accession number GSE146614. Source data for all graphs in this article are included in Supplementary Data 2. Uncropped data for all blots and gels in this article are included in Supplementary Fig. 10 and 11.

Field-specific reporting

Please select the one below that is the best fit for your research. If you are not sure, read the appropriate sections before making your selection.

[x] Life sciences   [ ] Behavioural & social sciences   [ ] Ecological, evolutionary & environmental sciences

For a reference copy of the document with all sections, see nature.com/documents/nr-reporting-summary-flat.pdf

Life sciences study design

All studies must disclose on these points even when the disclosure is negative.

Sample size	Sample size are described in figure legends.
Data exclusions	No data was excluded from the analysis.
Replication	All attempts replicated similar results. We have provided protocol on methods section.
Randomization	No randomization was used in this study.
Blinding	No blinding was done in this study.

Reporting for specific materials, systems and methods

We require information from authors about some types of materials, experimental systems and methods used in many studies. Here, indicate whether each material, system or method listed is relevant to your study. If you are not sure if a list item applies to your research, read the appropriate section before selecting a response.

Materials & experimental systems

n/a	Involved in the study	
[ ]	[x]	Antibodies
[ ]	[x]	Eukaryotic cell lines
[x]	[ ]	Palaeontology and archaeology
[ ]	[x]	Animals and other organisms
[x]	[ ]	Human research participants
[x]	[ ]	Clinical data
[x]	[ ]	Dual use research of concern

Methods

n/a	Involved in the study	
[x]	[ ]	ChIP-seq
[x]	[ ]	Flow cytometry
[x]	[ ]	MRI-based neuroimaging

Antibodies

Antibodies used	All the antibodies are commercial available, and the name of supplier and cataloger number are described in Supplementary table 5.
Validation	Validation is available on the manufacturer's website for use in mouse and human samples.

Eukaryotic cell lines

Policy information about cell lines

Cell line source(s)	Mouse ES cell (C57BL/6J strain)
Authentication	None of the cell lines used were authenticated.
Mycoplasma contamination	Cell lines were not tested for mycoplasma contamination.
Commonly misidentified lines (See ICLAC register)	The cell lines used in this study are not in the commonly misidentified lines list.

Animals and other organisms

Policy information about studies involving animals; ARRIVE guidelines recommended for reporting animal research

Laboratory animals	Generation of DYRK2-deficient mice are described in Result, Methods, and Supplementary information.<br>We mainly focus on the mice at age of E10.5, E11.5, E14.5, and E18.5.

Wild animals	The study did not involve wild animals.
Field-collected samples	The study did not involve samples collected from the field.
Ethics oversight	All animal experiments were approved by the Animal Care and Experimentation Committee of Gunma University and Institutional Animal Care and Use Committee of Jikei University.

Note that full information on the approval of the study protocol must also be provided in the manuscript.
